# Tempering in Glass Tubes

**Published:** 1900-03

**Authors:** Safford S. Perry

**Affiliations:** New York


### Domestic Correspondence.

### TEMPERING IN GLASS TUBES.

To the Editor:

Sir,—In the January number of the International Dental Journal, page 48, Dr. Howe, discussing Dr. Palmer's description of the use of Swiss broaches before the New York Institute of Stomatology, October 3, said, "In regard to tempering fine broaches, I would like to call attention to the method invented or devised by Dr. Meriam, who has tempered needles and other fine instruments in glass test-tubes. I have never known of any way by which delicate instruments could be tempered as successfully.

"The description of Dr. Meriam's method was published in connection with a paper on making crowns in the International Dental Journal for March of this year. I would advise all who are interested to read it and try his method."

In a paper on "Methods of Root Canal Filling," published in the September number of the International Dental Journal, 1895, page 530, in describing the tempering of Swiss broaches, I said, "I place a dozen or two of them in a glass tube and draw the temper to a deep blue, over an alcohol lamp or a Bunsen burner. The glass protects them from currents of cold air, allows them to cool slowly, and enables one to see the color of the steel."

This method was original with me, and was in use several years before it was described.

This is a trifling matter, but I am sure both Drs. Howe and Meriam will be glad to have their attention called to the published record.

Yours very truly,

Safford S. Perry.

New York, January 29, 1900.

